# Abnormal static and dynamic functional network connectivity in stable chronic obstructive pulmonary disease

**DOI:** 10.3389/fnagi.2022.1009232

**Published:** 2022-10-17

**Authors:** Fuqiu Tang, Lan Li, Dechang Peng, Jingjing Yu, Huizhen Xin, Xuan Tang, Kunyao Li, Yaping Zeng, Wei Xie, Haijun Li

**Affiliations:** ^1^Medical Imaging Center, The First Affiliated Hospital of Nanchang University, Nanchang, China; ^2^Department of Infection Management, Jiangxi Provincial Maternal and Child Health Hospital, Nanchang, China; ^3^PET Center, The First Affiliated Hospital of Nanchang University, Nanchang, China; ^4^Department of Respiratory and Critical Care, The First Affiliated Hospital of Nanchang University, Nanchang, China

**Keywords:** chronic obstructive pulmonary disease, dynamic, functional connectivity, cognitive impairment, independent component analysis

## Abstract

**Objective:**

Many studies have explored the neural mechanisms of cognitive impairment in chronic obstructive pulmonary disease (COPD) patients using the functional MRI. However, the dynamic properties of brain functional networks are still unclear. The purpose of this study was to explore the changes in dynamic functional network attributes and their relationship with cognitive impairment in stable COPD patients.

**Materials and methods:**

The resting-state functional MRI and cognitive assessments were performed on 19 stable COPD patients and 19 age-, sex-, and education-matched healthy controls (HC). We conducted the independent component analysis (ICA) method on the resting-state fMRI data, and obtained seven resting-state networks (RSNs). After that, the static and dynamic functional network connectivity (sFNC and dFNC) were respectively constructed, and the differences of functional connectivity (FC) were compared between the COPD patients and the HC groups. In addition, the correlation between the dynamic functional network attributes and cognitive assessments was analyzed in COPD patients.

**Results:**

Compared to HC, there were significant differences in sFNC among COPD patients between and within networks. COPD patients showed significantly longer mean dwell time and higher fractional windows in weaker connected State I than that in HC. Besides, in comparison to HC, COPD patients had more extensive abnormal FC in weaker connected State I and State IV, and less abnormal FC in stronger connected State II and State III, which were mainly located in the default mode network, executive control network, and visual network. In addition, the dFNC properties including mean dwell time and fractional windows, were significantly correlated with some essential clinical indicators such as FEV_1_, FEV_1_/FVC, and c-reactive protein (CRP) in COPD patients.

**Conclusion:**

These findings emphasized the differences in sFNC and dFNC of COPD patients, which provided a new perspective for understanding the cognitive neural mechanisms, and these indexes may serve as neuroimaging biomarkers of cognitive performance in COPD patients.

## Introduction

Chronic obstructive pulmonary disease (COPD) is one of the most common incurable pulmonary diseases which is characterized by persistent airflow limitation and some respiratory symptoms such as chronic cough, sputum, chest tightness, and dyspnea upon exertion (Rabe and Watz, 2017). According to a wide-ranged representative COPD epidemiology research in 2018, the estimated overall prevalence of COPD within adults aged 40 years or older in China was 13.6%, and among them, 43.7% had reached moderate COPD (Level 2) and above, which means that COPD has gradually become a major public-health problem compared to before (Fang et al., 2018). Studies have shown that COPD could cause a lot of comorbidities on different organs and systems, such as hypertension, osteoporosis, diabetes, brain dysfunction, mental illness, and so on (Vestbo et al., 2013). Among these comorbidities of COPD, cognitive impairment is recognized as one of the most serious diseases and most overlooked extra-pulmonary symptoms, for it would not only lead to the decreased quality of life, moreover, it might have adverse effects on the respiratory rhythm and frequency, which may lead to the deterioration of disease condition, if preventive measures couldn’t be taken timely (Yohannes et al., 2017). Thus, illustrating the internal neural mechanisms between COPD and cognitive impairment is of great importance. Hypoxia/hypercarbia and cognitive dysfunction in COPD patients are related to some extent, which might become one of the possible pathophysiological mechanisms (Parekh et al., 2005; Ortapamuk and Naldoken, 2006). However, the specific pathogenesis between cognitive impairment and COPD is still a problem to be researched and solved.

Due to successive airflow limitation of COPD, the brain cannot receive sufficient oxygen to a certain degree, causing inevitable changes in spontaneous brain activity which results in a series of elaborate metabolic abnormalities (Zhang et al., 2016). On account of this, the functional MRI, based on the blood oxygenation, provides us with an effective platform to study the relation between COPD and cognitive dysfunction (Glover, 2011). And in particular, the resting-state fMRI, the hotspot of neuroimaging and brain function research in recent years, can become a convenient and advanced tool to explain the pathogenesis of the relation without given stimulus or tasks (Smitha et al., 2017). Many scholars have devoted themselves to using fMRI to research the cognitive disorder before. For instance, a previous study, using the seed-based functional connectivity (FC) analyses, found decreased FC mainly in the visual network and frontoparietal network, which was then confirmed to be positively correlated with cognitive function assessed with MoCA scale by precise correlational analyses (Wang W. et al., 2020). Furthermore, another study about spontaneous neural activity in COPD patients found decreased local spontaneous activity in the left basal ganglia and novel temporal dynamic brain local activity alteration in the bilateral parahippocampal/hippocampal gyrus, more importantly, these alterations were linked with semantic-memory impairments which may be modulated by poor pulmonary function (Lv et al., 2021). And our recent study with amplitude of low-frequency fluctuation (ALFF) method have demonstrated that there were exceptional low-frequency oscillations amplitudes related with various brain physiological functions in some COPD patients’ given brain regions, and it would provide the neuroimaging with a new direction of exploration (Yu et al., 2021). Nevertheless, all the studies mentioned above just primarily focused on the traditional classical frequency band (0.01–0.1 Hz) and assumed that the functional network was static in the whole time, while ignoring the time variability.

Independent component analysis (ICA) is a data processing method suitable for resting-state fMRI, which could decompose the resting-state fMRI data into multiple brain networks without assuming in advance and further analyze these resting-state networks (Hu et al., 2019; Seewoo et al., 2021; Li et al., 2022). The ICA is an advanced data-driven approach, which evaluates the whole brain data and then separate it into individual components, enabling us to conduct in-depth observational studies of brain connectivity (van den Heuvel et al., 2009; Vargas et al., 2013). Some previous studies have already applied it to some related diseases, such as acute thyrotoxic myopathy (Li et al., 2022), attention deficit and hyperactivity disorder (Kumar et al., 2021), juvenile absence epilepsy (Parsons et al., 2020), high myopia (Ji et al., 2022), and so on. Our brains must dynamically integrate, coordinate, and respond to all internal and external stimuli so that we could feel, remember, think, and correlate with others (Hutchison et al., 2013). A growing number of research also point out that our brain functional network connections could be changeable with time going by Hutchison et al. (2013), Meier et al. (2016). Some scholars have proposed that the dFNC could reflect transient and recurrent whole-brain temporal coupling patterns (Wens et al., 2019), revealing the neural mechanism, and it already has been used in a variety of neuropsychiatric diseases, such as Parkinson’s disease (Fiorenzato et al., 2019), Major depressive disorder (Zhi et al., 2018), Sleep deprivation (Li C. et al., 2020), and so on. However, there is no report on using ICA approach to research the cognitive impairment caused by COPD at present. Therefore, through combining ICA and dynamic FC, it may help us better study the interaction of COPD and cognitive impairment.

To sum up, according to previous studies, we made the hypothesis that there might be temporal variability in brain network connectivity in COPD patients which is associated with clinical assessments such as cognition and memory. With regards to this, we used the ICA method to extract and detect different resting-state networks firstly. Then, the dynamic FC analysis was performed through a sliding-window time approach and the *k*-means clustering algorithm (Oh et al., 2021). At the same time, we compared the differences between the COPD patients and the HC groups in the temporal variation of dFNC. Eventually, we explored the relationship between the temporal variability and cognitive function, as well as other clinical assessments.

## Materials and methods

### Subjects

All 19 stable COPD patients and 19 age-, sex-, and education-matched healthy controls (HC) in this research were recruited in the Respiratory Department of the First Affiliated Hospital of Nanchang University (Nanchang, China) from December 2017 to May 2018. We determined the diagnostic criteria and classification on the basis of the Global Initiative for Chronic Obstructive Lung Disease (GOLD) guidelines from 2017 (Vogelmeier et al., 2017). All the patients were diagnosed with COPD and they were in stable state with no exacerbations during the past 8 weeks or after therapy by pulmonary function tests according to GOLD guideline (Vogelmeier et al., 2017). All individuals underwent a rigorous and detailed clinical history interview, a physical examination, a blood gas analysis and a pulmonary function test. In addition, to receive a more persuasive sample, we made a series of exclusion criteria, including: (1) obstructive sleep apnea syndrome or insomnia; (2) mental or neurological disorders like epilepsy; (3) brain damage; (4) severe cardiovascular diseases; (5) history of drugs and/or alcoholism; (6) comorbidities such as diabetes, anemia, and other major diseases; (7) the Montreal Cognitive Assessment (MoCA) and the Mini-Mental State Examination (MMSE) evaluations could not be completed; (8) participants with MRI contraindications were also excluded, such as claustrophobia, metallic implants in the body, and so on.

### Arterial blood gas analysis

We used the Stat Profile Critical Care Xpress (Nova Biomedical, Waltham, MA, USA) to detect some essential parameters of arterial blood gas, including arterial partial pressure of oxygen (PaO_2_), arterial partial pressure of carbon dioxide (PaCO_2_), the oxyhemoglobin saturation (SaO_2_), and pH (negative logarithm of hydrogen ion concentration in a standard volume of arterial blood sample).

### Pulmonary function test

The main indicators of lung function consisted of the following components: the forced expiratory volume in the first second (FEV_1_), the forced vital capacity (FVC), and the FEV_1_/FVC. These indexes were tested with the use of a dry spirometer device (Erich Jaeger GmbH, Hoechberg, Germany) after inhaling an appropriate dose of bronchodilator. Based on the GOLD (Vogelmeier et al., 2017), in COPD patients, the FEV_1_/FVC is less than 0.7 after inhalation of bronchodilators, and then we classified airflow limitation according to the magnitude of the drop in FEV_1_: the FEV_1_ ≥ 80% predicted were classified as mild COPD (Level 1), those with 50% ≤ FEV_1_ < 80% predicted were classified as moderate COPD (Level 2), those with 30 ≤ FEV_1_ < 50% predicted were classified as severe COPD (Level 3), and those with FEV_1_ < 30% predicted were classified as extremely severe COPD (Level 4) (Vogelmeier et al., 2017).

### Cognitive assessments

All participants conducted a series of cognitive assessments including the MMSE (Folstein et al., 1975) and the MoCA (Nasreddine et al., 2005). The MMSE and the MoCA were mainly used to test the common brain cognitive functions such as attention and naming, and the latter assessment consists of the following eight aspects: visuospatial and executive function, naming, memory, attention, language, abstraction, and orientation (Nasreddine et al., 2005). An MMSE score ≤ 26 or a total MoCA score < 26 indicates defective cognitive function (Folstein et al., 1975; Nasreddine et al., 2005).

### MRI data acquisition

All the MRI data were collected on 3.0 Tesla MR scanners (Siemens, Erlangen, Germany) with 8-channel head coils at the Department of Radiology of the First Affiliated Hospital of Nanchang University. All participants were asked to keep still, and keep their eyes closed but not to fall asleep or think about anything during the MRI scan. Foam pads were used to reduce head movements, and earplugs were used to decrease the noise. First, conventional axial T2-weighted imaging [repetition time (TR) = 4000 ms, echo time (TE) = 113 ms, thickness = 5 mm, gap = 1.5 mm, FOV = 220 mm × 220 mm, slices = 19] and axial T1-weighted imaging [TR = 250 ms, TE = 2.46 ms, thickness = 5 mm, gap = 1.5 mm, field-of-view (FOV) = 220 mm × 220 mm, slices = 19] were performed. Then, high-resolution three-dimensional T1-weighted images were obtained using a brain volume sequence (TR = 1900 ms, TE = 2.26 ms, thickness = 1.0 mm, gap = 0.5 mm, FOV = 250 mm × 250 mm, matrix = 256 × 256, flip angle = 9°, 176 sagittal slices). Finally, resting-state fMRI data were collected using an echo-planar imaging sequence with the following parameters: TR = 2000 ms, TE = 30 ms, flip angle = 90°, FOV = 230 mm × 230 mm, matrix = 64, thickness = 4 mm, gap = 1.2 mm. Each brain volume consisted of 30 axial sections, and each functional run comprised 240 volumes.

### Functional MRI data preprocessing

All the images were checked using MRIcro software,^1^ and then they were reviewed by two senior radiologists to check out, in order to prevent any emergencies before data preprocessing, for example, lacking of data. Based on MATLAB2018b (Mathworks, Natick, MA, USA) software, Statistical Parametric Mapping (SPM12^2^) and Data Processing and Analysis for Brain Imaging (DPABI^3^) were used for images preprocessing. The main images preprocessing steps were as follows: first, converted DICOM format to NII format; second, removed the first 10 time points in order to reach a stable signal state and ensure that all participants were fully acclimated to the scanning environment; after that, for the remaining volumes, we conducted slice timing and three-dimensional head motion correction to reduce the influence of image acquisition time and head motion on the data (The head motion correction standard: the maximum rotation angle is less than 2° or the maximum displacement distance in any direction is less than 2 mm); then, three-dimensional T1-weighted images were segmented into white matter, gray matter, and cerebrospinal fluid with the Diffeomorphic Anatomical Registration Through Exponentiated Lie algebra (DARTEL); later, the images were normalized to the standard Montreal Neurological Institute (MNI) template and resampled to 3 mm × 3 mm × 3 mm voxels; finally, the images were performed spatial smoothing with a Gaussian kernel of 6 mm full width at half maximum. Besides, we used a linear regression with the friston 24 parameter (6 head motion parameters, 6 head motion parameters one time point before, and the 12 corresponding squared items) (Friston et al., 1996), cerebrospinal fluid signals and white matter signals as interference variables from the rest of the data. See our previous study for more details (Li H. et al., 2020; Yu et al., 2021).

### Independent component analysis and resting-state networks identification

**FIGURE 1:**
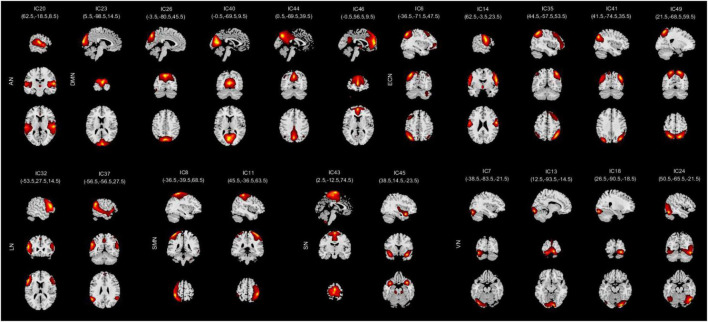
Spatial independent component analysis (ICA) method was used to identify independent components (*n* = 21). Divide the independent component spatial maps into seven functional networks according to their anatomical and functional properties, namely, AN, DMN, ECN, LN, SMN, SN, and VN. AN, auditory network; DMN, default mode network; ECN, executive control network; LN, language network; SMN, sensorimotor network; SN, salience network; VN, visual network.

After fMRI data processing, we used the GIFT software to conduct the independent component analysis (ICA) with the aim of turning the data into different brain functional networks. The principal component analysis (PCA) was carried out to achieve data-dimensionality reduction at the individual level and we got 120 ICs totally. Then, the data were reduced into 100 ICs with the help of expectation-maximization (EM) algorithm (Do and Batzoglou, 2008). After that, the Infomax ICA algorithm in ICASSO (Himberg et al., 2004; Kumar et al., 2021) was conducted for 10 times and the aggregate spatial maps were then formed. Eventually, we made group ICA to back-reconstruct the time courses and spatial maps of individual subjects (Wang C. et al., 2020). Among these ICs, we also had to ensure whether the peak activation coordinates were located in gray matter or not, and whether the time courses were dominated by low frequency vibrations. Based on previous studies (Shirer et al., 2012; Allen et al., 2014; Damaraju et al., 2014), we identified 21 significant components as RSNs and they were classified into seven RSNs according to the spatial correlation values between ICs and the template, including auditory network (AN), default mode network (DMN), executive control network (ECN), language network (LN), sensorimotor network (SMN), salience network (SN), and visual network (VN). As shown in Table 1 and Figure 1.

**TABLE 1:** Peak coordinates of the ICs.

IC regions	*T*max	Peak coordinate		
		
		X	Y	Z
**Auditory network**				
IC20 Bi Superior Temporal Gyrus	18.9	62.5	–18.5	8.5
**Default mode network**				
IC23 Bi Cuneus	17.3	5.5	–98.5	14.5
IC26 Precuneus	23.7	–3.5	–80.5	45.5
IC40 Precuneus	23.1	–0.5	–69.5	9.5
IC44 Posterior Cingulate Cortex	25.8	0.5	–69.5	39.5
IC46 Medial Prefrontal Cortex	23.3	–0.5	56.5	9.5
**Executive control network**				
IC6 L Lateral Occipital Cortex	24.2	–36.5	–71.5	47.5
IC14 Bi Middle Frontal Gyrus	18.0	62.5	–3.5	23.5
IC35 R Lateral Occipital Cortex	22.9	44.5	–57.5	53.5
IC41 Bi Lateral Occipital Cortex	25.6	41.5	–74.5	35.5
IC49 Bi Superior Parietal Lobule	20.3	21.5	–68.5	59.5
**Language network**				
IC32 L Inferior Frontal Gyrus	21.5	–53.5	27.5	14.5
IC37 L Angular Gyrus	17.1	–56.5	–56.5	27.5
**Sensorimotor network**				
IC8 L Pre-central/Post-central Gyrus	18.8	–36.5	–39.5	68.5
IC11 R Pre-central/Post-central Gyrus	16.4	45.5	–36.5	63.5
**Salience network**				
IC43 Dorsal Anterior Cingulate Cortex	22.6	2.5	–12.5	74.5
IC45 Bi Insular	21.6	38.5	14.5	–23.5
**Visual network**				
IC7 L Fusiform	12.8	–38.5	–83.5	–21.5
IC13 L Lingual	14.1	12.5	–93.5	–14.5
IC18 R Lingual	15.6	26.5	–90.5	–18.5
IC24 R Fusiform	15.4	50.5	–65.5	–21.5

ICs, independent components; L, left; R, right; Bi, bilateral.

### Static functional network connectivity analysis

We used the Mancovan toolbox in GIFT to compute the correlations between any two ICs time courses for each participant after ICA analysis. Then, the sFNC was acquired by computing the Pearson’s correlation coefficient between each summary time course and every other summary time course, thus generating a 21 × 20 matrix for each participant. Finally, we used a general linear model (GLM) (Poline and Brett, 2012), with age and education as nuisance covariates to determine the mean FNC of all subjects and which pair of FNCs was significantly different between all COPD patients and HC. The significance threshold was set at *p* < 0.01, false discovery rate (FDR) correction.

### Dynamic functional network connectivity analysis

We used a sliding time window approach to capture the dFNC. Since there was currently no formal consensus regarding the window length, according to former studies (Díez-Cirarda et al., 2017; Zhi et al., 2018), we made the window size set to 22 TRs and a Gaussian (σ = 3 TRs) and steps of 1 TR, and then constructed a series of FNC matrices. After that, the *k*-means clustering algorithm was performed on the FNC matrices and we determined the optimal value of *k* (Malhi et al., 2019). Later, the estimate clusters were computed in the standard dFNC matrices using gap and silhouette statistic (resulting in four states). Then the three dFNC indicators were calculated: (1) fractional windows (the percentage of time spent in each state out of the total time); (2) mean dwell time (the average length of time the subjects spent in a certain state); and (3) number of transitions (the number of times a subject switched between different states).

### Statistical analysis

The IBM SPSS 19.0 software was used to investigate the differences in clinical information between the COPD patients and HC. Chi-square tests were used in categorical variables, while the independent two sample *t*-tests were used for continuous variables and *p* < 0.05 was recognized statistically significant. For FNC, two sample *t*-tests were used to compare the three temporal properties of dFNC in four states, and *p* < 0.05 was recognized statistically significant. Moreover, the two sample *t*-tests was used to compare connectivity strength in each state between COPD and HC, with a significance threshold of *p* < 0.01 (FDR corrected). The dFNC values were used to evaluate correlations with clinical assessment scores using Pearson’s correlation analysis, and *p* < 0.05 was recognized statistically significant.

## Results

### Demographic and clinical data results

The demographic and neuropsychological characteristics of both groups are summarized in Table 2. There were no obvious differences between the COPD patients and HC in age, sex, and education. Despite this, we still found decreased scores in PaO_2_, SaO_2_, FVC, FEV_1_, FEV_1_/FVC, MMSE, and MoCA in COPD patients by contrast with HC, while obviously higher scores being exhibited for the PaCO_2_ and pack-years in COPD group. Furthermore, the intracranial volume and respiratory rates revealed no significant differences between the two groups.

**TABLE 2:** Demographic and clinical characteristics of COPD and HC.

Characteristic	COPD (*N* = 19)	HC (*N* = 19)	*P*-value
Age (years)	62.7 ± 5.9	62.3 ± 6.3	0.896
Male/Female (*N*)	14/5	14/5	1.000
Education (years)	5.5 ± 3.2	6.2 ± 2.7	0.453
Disease duration (years)	4.5 ± 5.6	/	/
Pack-years	27.9 ± 20.5	8.3 ± 6.5	<0.001
SaO_2_ (%)	95.5 ± 2.6	98.2 ± 1.8	0.007
PaO_2_ (mm Hg)	82.6 ± 16.5	98.1 ± 19.6	<0.001
PaCO_2_ (mm Hg)	49.3 ± 8.0	38.5 ± 4.2	<0.001
Respiratory rate (times/min)	19.5 ± 0.6	18.3 ± 1.2	0.654
FVC (% predicted)	67.5 ± 19.9	96.7 ± 15.4	<0.001
FEV_1_ (% predicted)	46.1 ± 20.6	97.1 ± 16.6	<0.001
FEV_1_/FVC (%)	55.8 ± 16.3	81.2 ± 8.3	<0.001
MMSE	22.4 ± 3.6	27.3 ± 2.2	<0.001
MoCA	18.4 ± 4.3	26.6 ± 3.2	<0.001
Intracranial volume (cm^3^)	1540.20 ± 110.49	1541.79 ± 99.17	0.915

COPD, chronic obstructive pulmonary disease; HC, health controls; SaO_2_, blood oxygen saturation; PaO_2_, partial pressure of oxygen; PaCO_2_, arterial partial pressure of carbon dioxide; FVC, forced vital capacity; FEV_1_, forced expiratory volume in the first second; MMSE, Mini-mental State Examination; MoCA, Montreal Cognitive Assessment; *N*, number.

### Resting-state networks results

**FIGURE 2:**
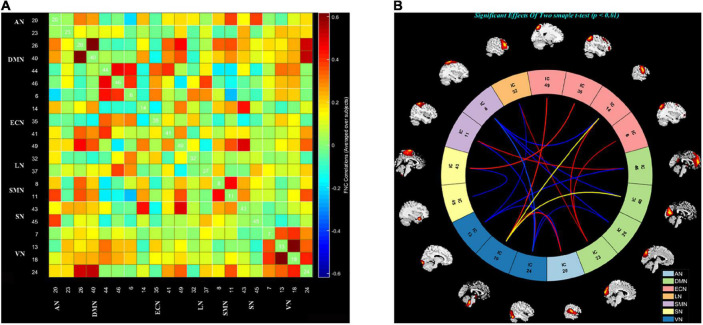
The static functional network connectivity results. **(A)** The average static functional network connectivity matrices of all subjects between ICs pairs were produced in entire resting state time courses. **(B)** The difference of static functional network connectivity between two groups in seven networks (two sample *t*-tests) (*p* < 0.01, FDR). AN, auditory network; DMN, default mode network; ECN, executive control network; LN, language network; SMN, sensorimotor network; SN, salience network; VN, visual network.

We grouped these individual components according to their respective anatomical and functional characteristics, thus identifying seven resting-state brain networks from the fMRI data after ICA, namely, AN (IC20); DMN (IC23 26 40 44 46); ECN (IC6 14 35 41 49); LN (IC32 37); SMN (IC8 11); SN (IC43 45); and VN (IC7 13 18 24). The averaged sFNC matrix between 21 ICs in seven networks of all subjects is shown in Figure 2A. Compared to HC, COPD patients exhibited significantly abnormal sFNC between DMN-ECN, DMN-LN, DMN-SMN, DMN-VN, ECN-SMN, ECN-VN, ECN-SN, VN-LN, AN-VN, VN-SMN, and within SN, VN. Detailed abnormal connection results are shown in Figure 2B.

### Dynamic functional network connectivity results

**FIGURE 3:**
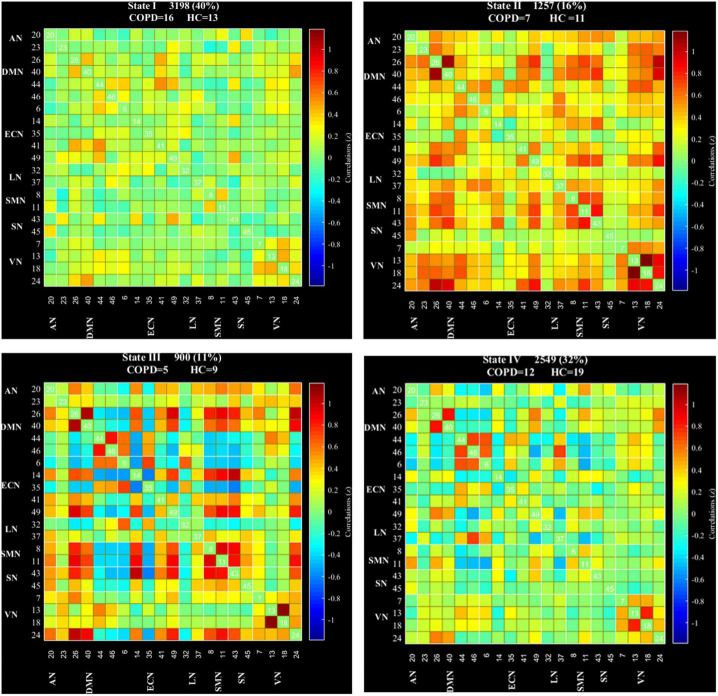
Dynamic functional network connectivity centroids of four states, the number of subjects, and the percentage of occurrence in each state. AN, auditory network; DMN, default mode network; ECN, executive control network; LN, language network; SMN, sensorimotor network; SN, salience network; VN, visual network; COPD, chronic obstructive pulmonary disease; HC, health controls.

**FIGURE 4:**
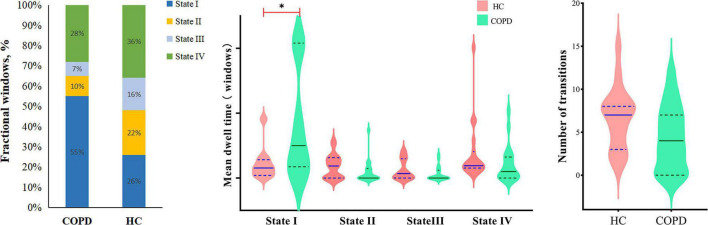
The relative proportion of the three dynamic functional network connectivity indicators (fractional windows, mean dwell time, number of transitions) in the two groups. COPD, chronic obstructive pulmonary disease; HC, health controls.

According to the estimate results of cluster status, the dFNC was clustered into four states by *k*-means clustering and the centroids of the four states are presented in Figure 3 (*k* = 4). It is important to note that the total number of subjects in each state is different, as not all subjects have four states. State I accounted for 40% (the largest occurrence frequency) and it contained 16 COPD patients and 13 HC, which was characterized by relatively much weaker connectivity among all the networks. State II accounted for 16% and State III accounted for 11% (the least occurrence frequency). The two states exhibited relatively stronger connections within networks. State IV occupied 32 percent, consisting of 12 COPD patients and 19 HC, which owned highly positive connections in DMN and VN, and relatively weaker or negative connections in other networks. Moreover, COPD patients showed significantly longer mean dwell time and higher fractional windows in State I (Tables 3, 4 and Figure 4), while no significant difference in these two attributes in other three states. However, no significant difference in number of transitions between two groups was found (Table 5 and Figure 4).

**TABLE 3:** Two sample *t*-tests of mean dwell time between the patients with COPD and HC.

State	COPD (Mean ± SD)	HC (Mean ± SD)	*t*-value	*P*-value
I	79.9 ± 83.1	22.8 ± 26.0	**2.673**	0.012
II	9.3 ± 17.6	17.7 ± 18.3	–1.553	0.13
III	6.4 ± 11.1	12.3 ± 15.5	–1.587	0.123
IV	21.3 ± 28.5	37.8 ± 46.4	–0.741	0.464

COPD, chronic obstructive pulmonary disease; HC, health controls; SD, standard deviation. The bold values indicate statistical significance.

**TABLE 4:** Two sample *t*-tests of fractional windows between the patients with COPD and HC.

State	COPD (Mean ± SD)	HC (Mean ± SD)	*t*-value	*P*-value
I	0.55 ± 0.38	0.26 ± 0.27	**2.859**	0.007
II	0.10 ± 0.18	0.22 ± 0.26	–1.437	0.159
III	0.07 ± 0.15	0.16 ± 0.23	–1.355	0.184
IV	0.28 ± 0.34	0.36 ± 0.30	–1.318	0.196

COPD, chronic obstructive pulmonary disease; HC, health controls; SD, standard deviation. The bold values indicate statistical significance.

**TABLE 5:** Two sample *t*-tests of number of transitions between the COPD and HC.

	COPD (Mean ± SD)	HC (Mean ± SD)	*t*-value	*P*-value
Number of transitions	4.4 ± 3.7	6.7 ± 3.6	−1.946	0.06

COPD, chronic obstructive pulmonary disease; HC, health controls; SD, standard deviation.

**FIGURE 5:**
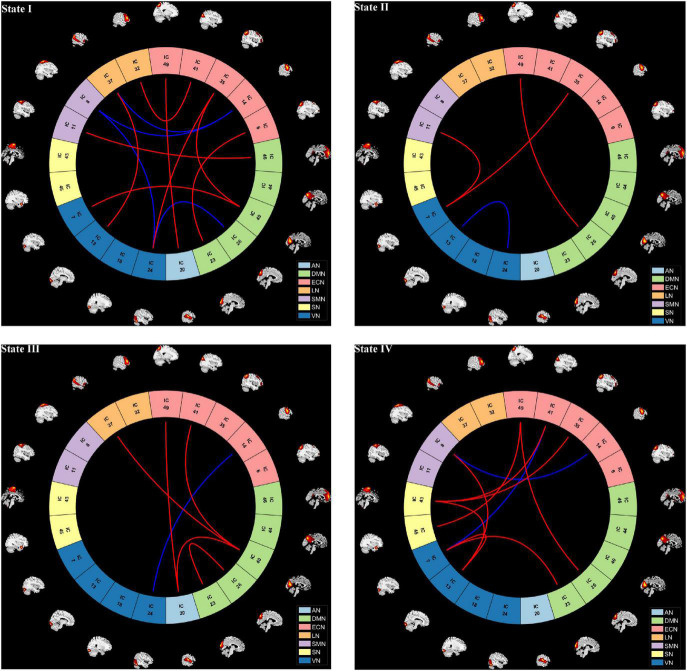
The visualization of dynamic functional network connection intensity differences in each state. Red line indicates higher functional connection strength in COPD compared to HC group, while the blue line represents reduction. AN, auditory network; DMN, default mode network; ECN, executive control network; LN, language network; SMN, sensorimotor network; SN, salience network; VN, visual network.

We further compared the strength of connections between the COPD and HC groups in four different states, and the results are shown in Figure 5. In State I, compared to HC, COPD patients showed stronger connections between ECN-DMN, ECN-AN, ECN-VN, ECN-LN, DMN-SMN, DMN-VN, VN-LN, and weaker connections between ECN-SMN, VN-SMN, DMN-VN. In State II, 3 stronger connections and 1 lower connection were found in COPD patients, including ECN-DMN, ECN-VN, SMN-VN, and within VN. Additionally, in State III, COPD patients exhibited 5 stronger connections and 1 lower connection, which were primarily located in ECN-DMN, ECN-AN, DMN-LN, and ECN-VN. Besides, abnormal connectivity between ECN, DMN, SMN, SN, and VN were also found in State IV. Overall, these abnormal functional connections mainly occurred in DMN, ECN, and VN.

### Correlation results

The correlation between the dFNC indicators and clinical performance in the COPD group was further analyzed. We found that the mean dwell time and fractional windows in some states were correlated with the clinical characteristics, especially FEV_1_, FEV_1_/FVC, PaCO_2_, and c-reactive protein (CRP). Detailed information is listed in the Table 6.

**TABLE 6:** Correlations between temporal properties of dFNC and clinical characteristics in COPD patients.

		MoCA	MMSE	ESS	FEV_1_	FVC	FEV_1_/FVC	PaO_2_	PaCO_2_	SaO_2_	pH	CRP
Fractional windows in State I	*r*	–0.072	0.039	0.106	–0.455	–0.256	–0.368	–0.233	0.383	–0.386	−0.507*	0.545*
	*p*	0.769	0.875	0.667	0.050	0.290	0.121	0.337	0.105	0.103	0.027	0.016
Fractional windows in State II	*r*	0.316	0.156	–0.029	0.725**	0.260	0.559*	0.291	−0.609**	0.440	0.496*	–0.104
	*p*	0.187	0.523	0.907	0.000	0.283	0.013	0.227	0.006	0.059	0.031	0.673
Fractional windows in State III	*r*	0.170	0.016	0.034	0.672**	0.057	0.491*	0.030	–0.368	0.309	0.445	–0.064
	*p*	0.487	0.948	0.889	0.002	0.815	0.033	0.904	0.121	0.198	0.056	0.795
Fractional windows in State IV	*r*	–0.091	–0.189	–0.242	0.135	0.216	0.264	0.027	–0.214	0.132	0.399	–0.392
	*p*	0.710	0.438	0.319	0.582	0.375	0.274	0.913	0.379	0.589	0.091	0.097
Mean dwell time in State I	*r*	0.087	0.168	0.239	−0.558*	–0.302	−0.556*	–0.323	0.405	–0.447	−0.514*	0.491*
	*p*	0.723	0.492	0.325	0.013	0.209	0.013	0.177	0.086	0.055	0.024	0.033
Mean dwell time in State II	*r*	0.331	0.166	–0.036	0.705**	0.258	0.545*	0.279	−0.637**	0.415	0.491*	–0.051
	*p*	0.167	0.497	0.885	0.001	0.287	0.016	0.247	0.003	0.077	0.033	0.836
Mean dwell time in State III	*r*	0.226	0.028	0.092	0.638**	0.026	0.466*	–0.011	–0.413	0.283	0.488*	–0.043
	*p*	0.353	0.910	0.707	0.003	0.917	0.044	0.966	0.079	0.240	0.034	0.863
Mean dwell time in State IV	*r*	–0.076	–0.151	–0.274	0.137	0.243	0.255	0.047	–0.212	0.154	0.409	–0.360
	*p*	0.757	0.536	0.257	0.577	0.316	0.291	0.849	0.383	0.529	0.082	0.130
Number of transitions	*r*	0.041	–0.114	–0.116	0.672**	0.267	0.729**	0.191	−0.600**	0.390	0.599**	–0.230
	*p*	0.868	0.641	0.636	0.002	0.269	0.000	0.434	0.007	0.098	0.007	0.344

dFNC, dynamic functional network connectivity; COPD, chronic obstructive pulmonary disease; HC, health controls; MoCA, Montreal Cognitive Assessment; MMSE, Mini-mental State Examination; FEV_1_, forced expiratory volume in the first second; FVC, forced vital capacity; PaO_2_, partial pressure of oxygen; PaCO_2_, arterial partial pressure of carbon dioxide; SaO_2_, blood oxygen saturation; pH, negative logarithm of hydrogen ion concentration in a standard volume of arterial blood sample; CRP, c-reactive protein.

*p < 0.05, **p < 0.01.

## Discussion

So far as we know, this is the first study to combine ICA and FNC to explore the changes in brain networks in COPD patients. In this study, the results have shown that both sFNC and dFNC of the COPD patients’ brain networks were altered. The COPD patients had longer fractional windows and longer mean dwell time in State I. And the changes of dFNC properties were linked to cognitive deterioration. In summary, these results could support the hypothesis that there is temporal variability in brain network connectivity and the abnormal sFNC and dFNC might have something to do with cognitive impairment in patients with COPD.

Compared to HC, significantly abnormal sFNC among all these resting-state networks in COPD patients could be seen. The ECN is responsible for initiation, planning, organization, and decision-making (Tuchscherer et al., 2010). The DMN, which is mainly responsible for social cognition, working memory, decision-making, and awareness (Buckner and DiNicola, 2019), consists of the discrete, bilateral and symmetrical cortical areas in the medial prefrontal cortex, posterior cingulate cortex, and precuneus (Raichle, 2015). In previous researches on neurological or psychiatric diseases like temporal lobe epilepsy and normal aging, the changes of ECN and DMN have been confirmed to be related to cognitive impairment to a certain extent (Chand et al., 2017; Zhang et al., 2020). Our results showed that the decreased sFNC of COPD patients were mainly concentrated on SMN, DMN, SN, and ECN. Simultaneously, the COPD group exhibited increased sFNC between DMN, ECN, SMN, and within VN. In addition, we also found that there are basically abnormal functional connections between the various networks, and the overall situation is in a messy state. All findings may represent that cognitive impairment in COPD patients is not only manifested in the impairment of lower-level perceptual brain networks, but also in various higher-level cognitive functions, especially those responsible for the DMN and ECN.

The loose and weak connections in State I, the meaning of inefficient functional integration and less flexible interaction, indicates that all kinds of advanced cognitive functions that should have been completed through the interaction between various brain regions cannot be performed well. And another point to note is that the fractional windows and mean dwell time increased significantly in State I. We speculated that it is the weak connectivity and the long mean dwell time in State I that leads to the poor connection and sparse interaction of related brain regions, which may be one of the pathogenesis of cognitive impairment in COPD patients. The incidence of the segregated State I was observed more frequently in COPD patients, which confirms our hypothesis further that the temporal characteristics are related to cognitive impairment indeed. Coincidentally, a study of cirrhosis and hepatic encephalopathy showed that patients spent significantly longer mean dwell time and fractional windows in State IV (the weakest FNC of all networks), and there was a significant correlation between these two properties and psychometric hepatic encephalopathy score, which is mainly related to cognitive function (Jiang et al., 2020). This is somewhat similar to what we have found. The common feature of State II and State III was that the FC between ECN and DMN is strong, while the results found patients had the least fractional windows and the shortest mean dwell time in these two states. We believed that it is this short stay that is not conducive to the communication and further causes cognitive impairment.

We compared the differences in the strength of dFNC across states and found that aberrant interactions were mainly manifested in these networks, DMN, ECN, and VN included. The activated brain regions in the DMN of COPD patients, were found to develop significantly different FC values, and the FC values were less than that of normal controls, in the meanwhile, the mutation of FC values was correlated well with cognitive function and pulmonary function (Hu et al., 2018). The ECN covers several medial-frontal areas, including anterior cingulate and paracingulate, and these brain areas correspond to several cognition paradigms, as well as action-inhibition, emotion, and perception-somesthesis-pain (Smith et al., 2009). Previous studies have pointed out that the ECN is prone to be active during cognitive tasks, and dysregulation of this network is thought to affect perception and corresponding sensory cortical activity, but during this period, the DMN is deactivated (Brewer et al., 2011; Hemington et al., 2016; Zou et al., 2021). That is to say, the relationship between DMN and ECN is considered to be mutually inhibiting (Zou et al., 2021). Our results exhibited an increase of the sFNC and dFNC between DMN and ECN, suggesting that it may be due to the abnormality of the SN, that leads to functional crosstalk between them. Therefore, the unbalanced state may let the internal stimulation tasks get disordered, and brain activities cannot be performed in a normal functional isolation state, resulting in a significant increase in the FC between them. Increased FC between ECN and DMN also has been found in many other cognition-related diseases, such as co-occurrence of schizotypy and obsessive-compulsive traits (Wang Y. M. et al., 2020) and Lennox-Gastaut syndrome (Warren et al., 2017).

Changes in visual information processing can cause cognitive impairment (Yener et al., 2014). Previous research also found that COPD patients showed decreased FC within the VN and it was positively correlated with the MoCA, language-domain score and attention-domain score (Wang W. et al., 2020). We observed a decrease in dFNC within the VN, the symbol of functional segregation, may become the reason why decreased visual resources could lead to cognitive impairment. We speculated that it may be due to the change in the volume of gray matter in the VN-related brain regions, resulting in a decrease in the interaction between VN and other networks, which further causes visual information to fully stimulate the corresponding higher-level networks in failure, thereby causing cognitive impairment. In our study, we found reduced FC between VN and ECN, DMN, and the results are consistent with our speculation.

The SN, consisting of the anterior insula and the anterior cingulate cortex, functions to segregate the most relevant among internal and external stimuli in order to guide behavior (Menon and Uddin, 2010). If we compare the brain networks to plenty of railways, the SN is the console on the railway, distinguishing the various stimuli, and then controlling the “trains” to reach different destinations through different railways. Our research showed that COPD patients are more prone to exhibit increased dFNC between SN and other networks, and decreased dFNC within VN. This would lead to a chaos in the interaction between SN and other networks, so that various internal stimuli can randomly activate the different brain networks in an unselective manner. Abnormal connectivity about SN also has previously been shown to be related with cognitive impairment, which is consistent with our results (Song et al., 2021). Thence, the misallocation and unequal allocation of corresponding cognitive resources could also be an explanation for why COPD patients suffer from cognitive impairment. In addition, the abnormal connections between several other networks were also found, including AN, LN, and SMN. Compared with the higher-level cognitive networks mentioned above, these are all lower-level cognitive networks. Many scholars have studied their association with cognitive impairment before and they are also more or less involved in the pathogenesis of cognitive impairment. For example, the dysexecutive factor in Parkinson’s disease was independently related to decreased connectivity in the SMN (Lang et al., 2019).

Furthermore, in our study, the correlation analysis on dFNC temporal properties and clinical characteristics in COPD patients showed that fractional windows, mean dwell time in States II and III were positively associated with FEV_1_ and FEV_1_/FVC, meaning that longer time spent in these states was related with lower lung COPD severity. On the contrary, we discovered negative correlation between the dFNC temporal characteristics in State I and some clinical indicators such as FEV_1_, FEV_1_/FVC and pH. Considering the high percentage on fractional windows and long mean dwell time in State I of COPD patients, this may be the reason why many COPD patients have poor lung function. And in State I, we also found positive correlation about CRP. High level of CRP, the early indicator of infectious or inflammatory conditions, could increase the risk of death in stable COPD patients (Clyne and Olshaker, 1999; Fermont et al., 2019). And a study found that chronic inflammation may contribute to neurodegenerative brain changes that underlie differences in cognitive ability in later life (Conole et al., 2021). Therefore, we have reasons to believe that increased CRP level is closely related to cognitive impairment in COPD patients.

Our study also has certain limitations and defects in some aspects. First, it was relatively limited just in 38 samples although this meets the requirements of statistics. Subsequent studies suggest a larger sample size based on our study. Second, the research was just based on ICA method and other measures like seed-based FC analysis and ALFF were not taken into account, and we only studied the resting-state networks extracted by ICA without considering the influence of other brain networks. Thus, the advantages and disadvantages of it and other methods in studying brain networks still need to be further explored. In addition, although all the participants were told not to think about anything and keep relatively still during the MRI scan, we still could not be sure if they actually made it and how much it would affect the results.

## Conclusion

On the whole, our study showed that both sFNC and dFNC of COPD patients have undergone significant changes which are associated with cognitive impairment. And there is temporal variability in brain network connectivity in COPD patients. These findings help us understand the underlying neural mechanisms of cognitive impairment in COPD patients, and the dFNC with its attributes might be used as biomarkers for assessment of cognitive impairment in COPD patients.

## Data availability statement

The original contributions presented in this study are included in the article/supplementary material, further inquiries can be directed to the corresponding author.

## Ethics statement

The studies involving human participants were reviewed and approved by Medical Ethics Committee of the First Affiliated Hospital of Nanchang University. The patients/participants provided their written informed consent to participate in this study.

## Author contributions

HL guided and designed the MRI experiment and analyzed the resting-state fMRI data. LL performed statistical analysis. FT and LL wrote the manuscript. JY, HX, XT, KL, YZ, and WX collected the resting-state fMRI data and clinical data. HL and DP reviewed and revised the manuscript. All authors contributed to the article and approved the submitted version.

## Footnotes

1
www.MRIcro.com

2
https://www.fil.ion.ucl.ac.uk/spm/software/spm12/

3
http://rfmri.org/dpabi
